# Wound healing and photoprotection properties of *Acanthus ebracteatus* Vahl. extracts standardized in verbascoside

**DOI:** 10.1038/s41598-024-52511-8

**Published:** 2024-01-22

**Authors:** Mayuree Kanlayavattanakul, Mattaka Khongkow, Nattaya Lourith

**Affiliations:** 1 School of Cosmetic Science, Mae Fah Luang University, Chiang Rai, 57100 Thailand; 2 Phytocosmetics and Cosmeceuticals Research Group, Mae Fah Luang University, Chiang Rai, 57100 Thailand; 3 National Nanotechnology Center (NANOTEC), National Science and Technology Development Agency, Pathum Thani, 12120 Thailand

**Keywords:** Biological techniques, Chemical biology

## Abstract

*Acanthus* spp. have been documented in traditional Thai herbal medicine and are applicable for the treatment of inflamed skin with wound healing property. Nonetheless, the scientific evidence necessary to prove the herb’s doctrine has not yet been revealed. Verbascoside-rich extracts of the herbal medicine *A. ebracteatus* Vahl., were therefore prepared. The extracts and verbascoside were examined for their wound healing abilities using a scratch assay with fibroblasts. The anti-inflammatory effect suppressing MMP-9 was assessed in cocultures of keratinocyte (HaCaT cells) and fibroblasts. The extracts significantly improved wound healing compared with the control (p < 0.001). The wound healing effect of the extracts significantly (p < 0.01) increased with increasing verbascoside content. It should be noted that the extract was significantly (p < 0.05) better than verbascoside at the same test concentration. The extracts were capable of protecting cocultures of HaCaT cells and fibroblasts from photodamage. The extracts significantly (p < 0.001) suppressed cellular MMP-9 secretion following UV exposure, showing a better effect than that of verbascoside (p < 0.01). *A. ebracteatus* extract is promising for wound healing and photoprotection, and a prominent source of verbascoside. Verbascoside-rich *A. ebracteatus* could be utilized for the development of innovative skin-care products.

## Introduction

The use of traditional medicine is a common practice in primary healthcare that has recently been increasing in its application and demand. The preference for medicinal herbs is prominent in regard to the advancement of extract standardizations and scientific background on their mechanisms and biological activities^1,2^. Various recipes are used for disease treatment and prevention. The use of natural treatments for wound healing has been of significant interest^3,4^.

Wound healing process is a complex cellular and molecular mechanisms that generally defied in 3 phases, i.e., inflammatory, proliferative and maturation phases. Wound healing phases are not represented distinctly but are overlap and continuous. The inflammatory responses secreted during the inflammatory phase protect the invading pathogens. On the means time, inflammatory mediators are involved in tissue degradation. Skin cells are proliferated in a high activity following cutaneous injury modulated by the growth factors such as fibroblast growth factor (FGF). Consequently, cell migration further forwards onto the wound bed restoring dermal barrier and function^3,5^. The sensitive balance between the diverse states of healing is crucial to tissue repairing following injury. Which, inflammation is needed to be balanced for the homeostasis for cellular proliferations and further maturated for a good quality of wound healing. Inflammation is therefore widely examined in cutaneous repairing study^5^. In contrary to different state relevant to extracellular matrices that are the skin compartments, which is sparely to be revealed. Matrix metalloproteinases (MMPs) are one of the important factors in wound healing downstream cell migration, proliferation, differentiation and lessen quality of the healing response^3^. Which, MMP-9 is the most frequently studied MMP in would healing process due to its high mechanistic connection and cellular remodeling^5^. MMP-9 is secreted with neutrophil activation by inflammatory signals. Further, the enzyme activates inflammatory mediators and blunts the inflammation^6^. In addition, MMP-9 was reported to delay wound healing by interference with re-epithelialization^7^. Thus, an inhibition against MMP-9 would of significant in wound healing process^8^.

**Figure 1:**
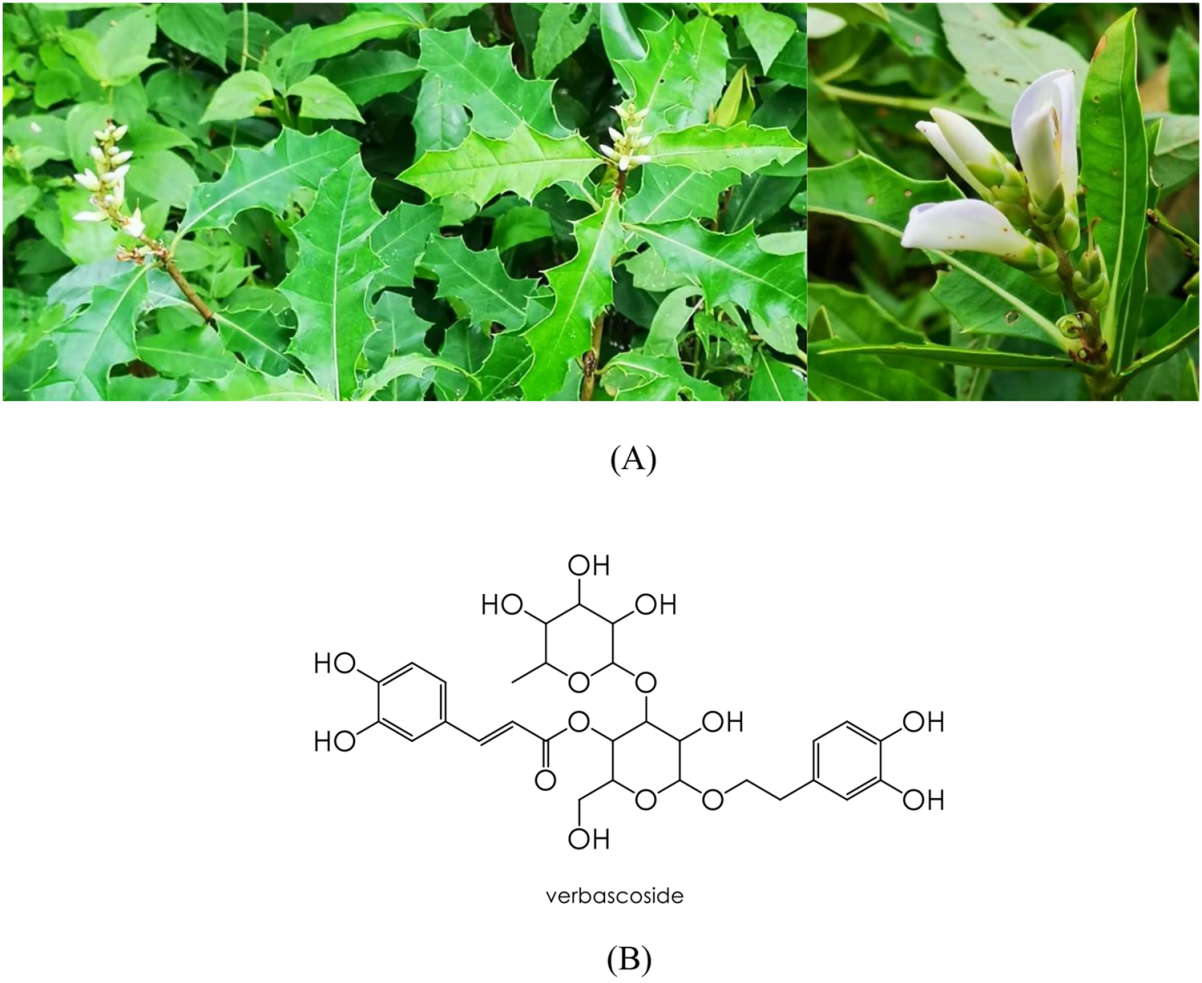
*Acanthus ebracteatus* (**A**) and verbascoside (**B**).

*Acanthus* spp. or sea holly have been documented in several Asian recipes. *A. ebracteatus* (Fig. 1A) has been included in a recipe that can improve the quality of life of cancer patients^9^. In addition, this herb has been documented in traditional Thai medicine for longevity and treating skin disorders^10,11^ including the recipes for wound healing^12^. Which, might be ruled by the herbal extract’s anti-inflammatory effects that were reported previously^13^. Accordingly, the herb is commercially cultivated and supplied for traditional drug stores in Thailand servings for the demand of the herb uses in traditional medicines.

Anti-inflammatory activity is an important therapeutic effect of natural active compounds for the care of skin wounds^4^. In particular, verbascoside (Fig. 1B), a caffeic acid glycoside that has been used for this purpose^3^. Verbascoside has been reported as a constituent of *Acanthus* spp., including *A. ebracteatus*^14,15^. Great interest has been shown in verbascoside as a natural active compound for the treatment of human diseases and disorders^16^ including wound healing^17^. Nonetheless, scientific-based proves of *A. ebracteatus* for skin disorders treatments abided with its traditionally claimed uses are sparely to be revealed.

In this study, extracts of the medicinal herb, *A. ebracteatus*, were prepared and standardized in terms of their verbascoside contents. The wound healing effects of the extracts and verbascoside were comparatively evaluated in fibroblasts. In addition, the activities of the extracts and verbascoside against MMP-9 were studied in cocultures of keratinocyte (HaCaT cells) and human dermal fibroblasts (HDF). The ethnopharmacological *A. ebracteatus* standardized in verbascoside is therefore proved upon its safety and efficacy in cell culture models, which is of challenge for an in vivo assessment in the future and additional working mechanisms besides its activity against MMP-9. Innovative topical products derived from the ethnobotany of folk remedies are challenged to be develop accordingly.

## Results

### Preparation of *A. ebracteatus* extracts standardized in verbascoside

**Figure 2:**
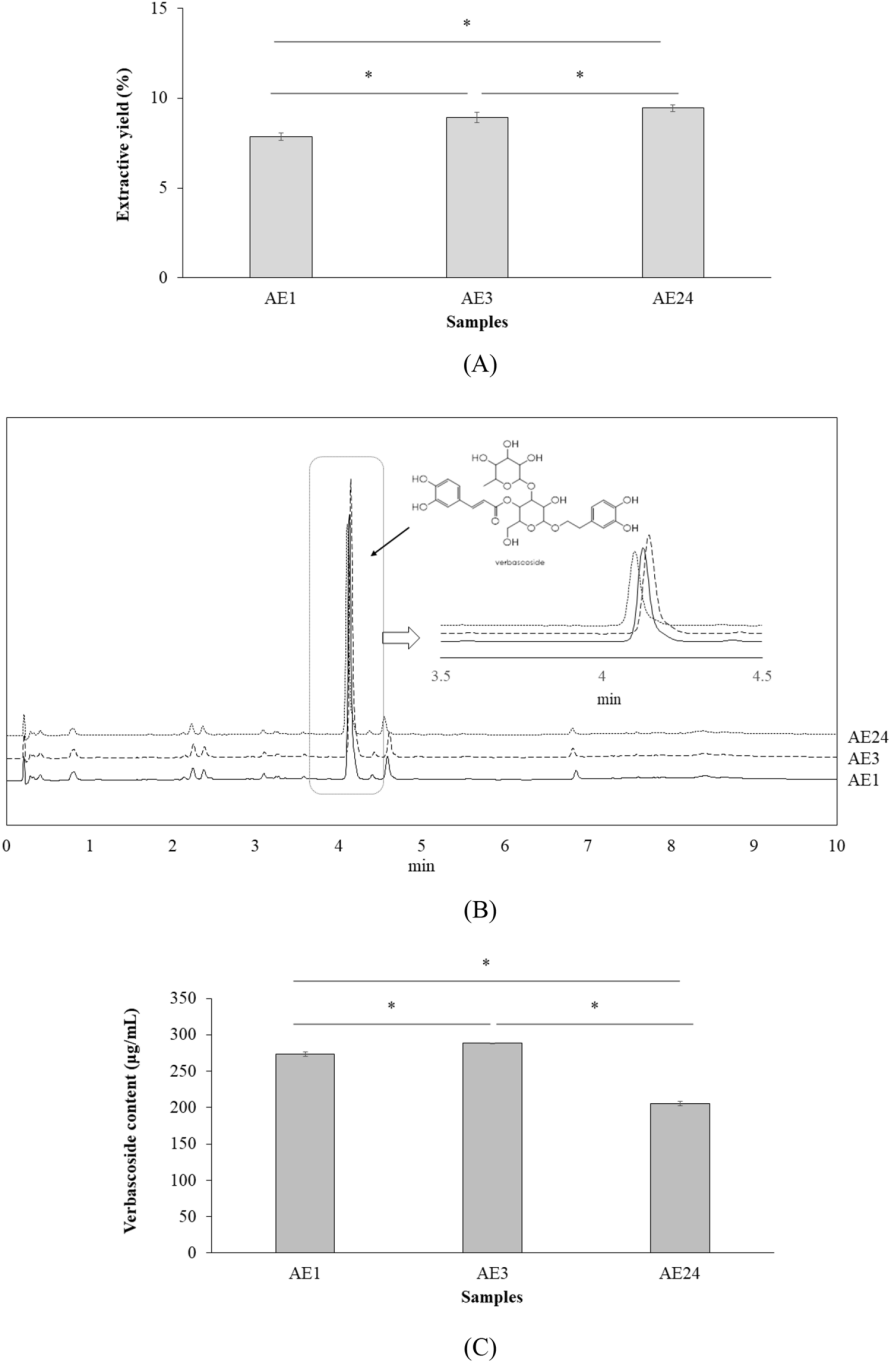
Extractive yields (**A**), representative of UPLC chromatograms (**B**) and verbascoside content of AEL extracts (**C**) (*p < 0.05).

*A. ebracteatus* leaves were macerated in 95% EtOH for different lengths of time, and longer extraction times significantly (p < 0.05) increased the extractive yield (Fig. 2A). Which, that of 24 h was greatest. Thereafter, the content of the phyto-active molecule of interest, verbascoside, was standardized by UPLC (Fig. 2B). The verbascoside content in AE3 was significantly (p < 0.05) higher than that in the extracts obtained at 1 and 24 h (Fig. 2C). Thereafter, the wound healing effect of each extract was assessed.

### Wound healing effect of the *A. ebracteatus* extracts

**Figure 3:**
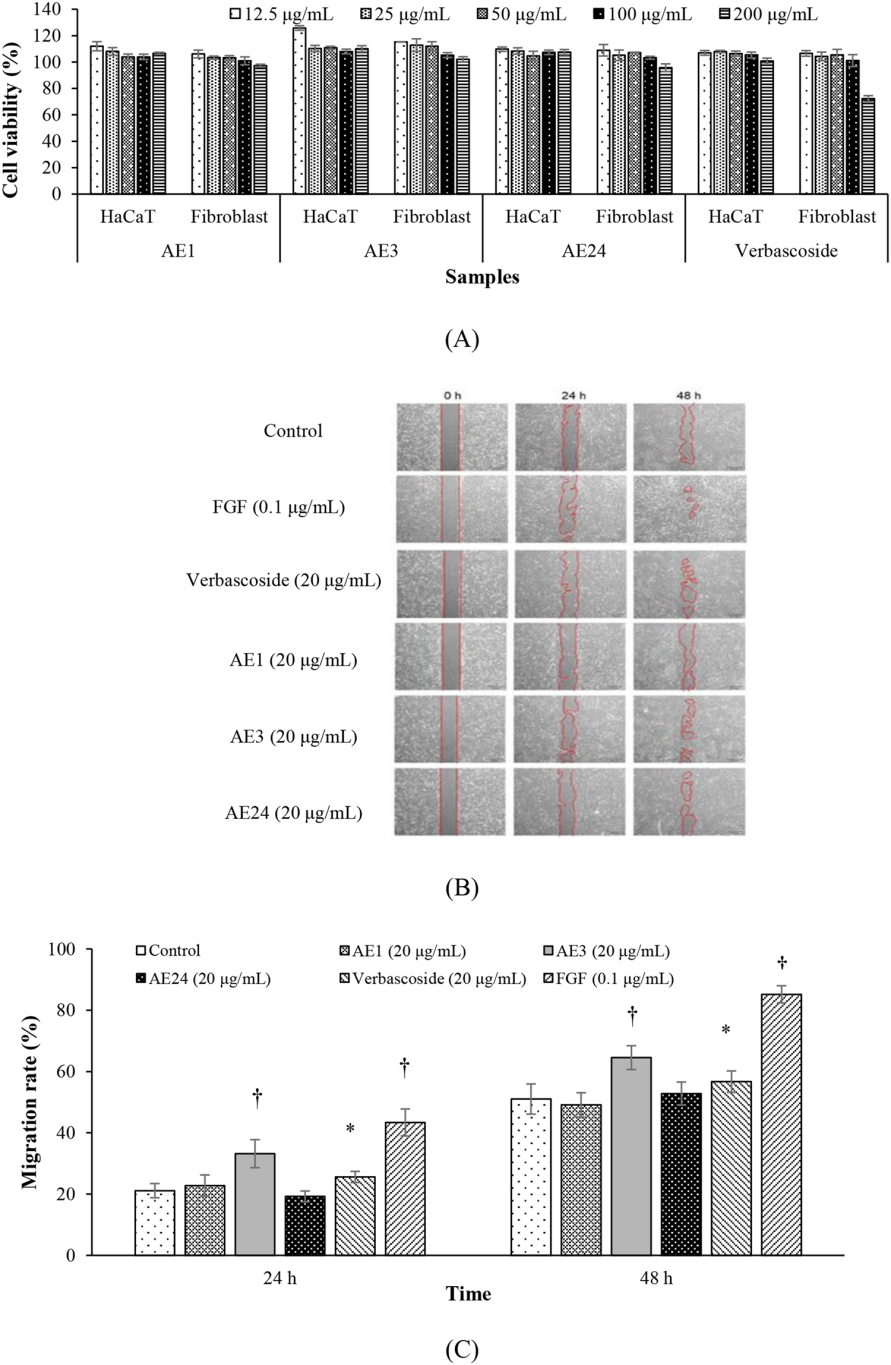
Safety assessments of the extracts in fibroblasts and HaCaT cells (**A**), representative images (**B**) and the cellular migration rate (**C**) of the scratch wound healing assay of cells treated with the samples at different time points (*p < 0.01, †p < 0.001).

Wound healing effect of the *A. ebracteatus* extracts standardized in verbascoside was challenged at the non-cytotoxic concentration towards fibroblasts (Fig. 3A) that cell viability of more than 80% was accounted as the non-cytotoxic one in addition to an observation on cell morphology, which visually identical with the control untreated group. All of the extracts were examined for their ability to stimulate fibroblast migration in a scratch assay. FGF, the benchmark active, exhibited the best ability to heal the wound (Fig. 3B), with a migration rate or wound closure^18,19^ of 43.37 ± 4.41% at 24 h, which was significantly (p < 0.001) better than that of the control untreated group. The wound healing property of FGF was pronounced after 48 h, giving a migration rate of 85.18 ± 2.81% (Fig. 3C). Among the extracts, the wound healing effect of AE3 was the best at all time intervals (33.19 ± 4.57% and 64.54 ± 3.89% at 24 and 84 h, respectively). AE1 and AE 24 showed weak wound healing activities and were slightly better than the control, but the difference was insignificant (p > 0.05). Verbascoside significantly (p < 0.01) improved wound closure compared with the control. It should be noted that AE3 was significantly (p < 0.05) better than its active component verbascoside.

### Inhibitory effect against MMP-9 of the *A. ebracteatus* extracts

**Figure 4:**
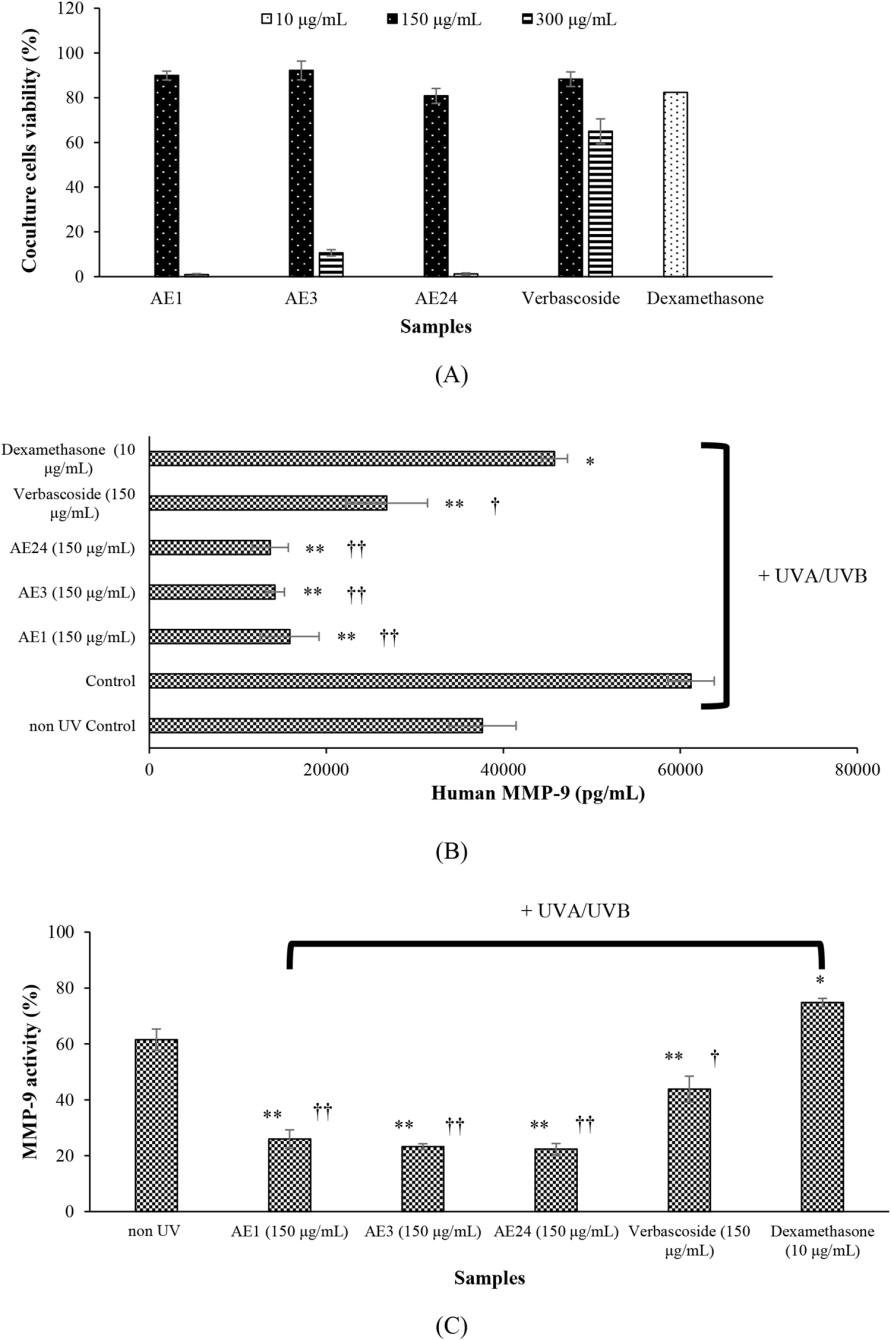
Safety assessments in coculture system of HaCaT cells and fibroblasts (**A**) of the extracts, measurement of MMP-9 in the cells treated with the samples (**B**) and activity of the samples against UV-induced MMP-9 expression (**C**) (*p < 0.01 and **p < 0.001 in a comparison with control, †p < 0.01 and ††p < 0.001 in a comparison with dexamethasone).

The activity of the extracts against MMP-9 was thereafter examined in cocultures of HaCaT and fibroblast cells. The cellular safety of the extracts was examined in HaCaT cells in addition to fibroblasts, individually. The extracts (12.5–200 µg/mL) were shown to be non-cytotoxic to the cells (Fig. 3A). The effects of the doses in the cellular safety range were thereafter assessed in the cocultures. Cytotoxicity was induced in the cocultures by UVA and UVB exposures, and the ability of the extracts to protect against photoaging was examined. The extracts and the extract’s active component, verbascoside, at 150 µg/mL were indicated to be non-cytotoxic and able to prevent photodamage in the cells as exhibited in Fig. 4A. At which, the benchmark active, dexamethasone was 10 µg/mL. The extracts were warranted on their safety in similar to the marker constituent determined, i.e., verbascoside. Thereafter, the biological activity on MMP-9 was observed at the samples’ safety concentrations. After UV exposures of the cocultures, the MMP-9 content was monitored (Fig. 4B). All of the AEL extracts significantly (p < 0.001) suppressed cellular MMP-9 secretion compared with the UV-exposed control cells, showing similar activity to that of verbascoside (p < 0.01) and the benchmark, dexamethasone (p < 0.001). The MMP-9 activity (%) following UV-induced expression was also thereafter calculated (Fig. 4C), and AE3 and AE24 comparatively (p > 0.05) inhibited MMP-9 activity. The dexamethasone was exhibited to suppress the content of MMP-9 to 74.80 ± 1.48%. Notably, the anti-MMP-9 activities of the extracts were superior over verbascoside (p < 0.001).

## Discussion

Sea holly has long been documented in longevity recipes in traditional Thai herbal medicine and is applicable for the treatment of inflamed skin^10,11,13^. In addition, the herb has been formulated in the recipes for wound healing^12^. Among these herbs, the white flower cultivar, *A. ebracteatus*, has been widely used rather than the purple cultivar, *A. illicifolius*, in accordance of its greater ethnopharmacological activities. *A. ebracteatus* is therefore commercially cultivated and supplied for traditional drug stores in Thailand. Nonetheless, the scientific evidence necessary to prove its traditional claims relevant to skin wound treatment is sparely to be addressed.

### Preparation of *A. ebracteatus* extracts standardized in verbascoside

*A. ebracteatus* is cultivated widely in Thailand. At which Nan Province is the major location supplied for traditional drug stores. The herb collected from the farm there was therefore examined in this presenting study. The herb was macerated in 95% EtOH in accordance with its traditional preparation by maceration or percolation in 95% EtOH. Which, that of 3 h extraction was exhibited as the most optimal time with the highest extractive yield (Fig. 2A). It should be noted that this presenting study gave better yields of the herbal extract than the traditional preparation by percolation for 5 days (5.25%)^13^, but lower than a maceration in EtOH for 3 days at the ratio between herb and solvent of 1:4 (w/v) that yielded 18.19 ± 0.75% of the extract^20^. That the source of the herb as per the herbal preparation practice prior to extraction relevant to the sample’s quality would co-contributed on the extractive yield. Thus, the maceration with a shorter time as per less volume of the solvent presented in this study shall be more feasible for the *A. ebracteatus* extract preparation practice.

Verbascoside has been reported as the major constituent of the herb^14,15^. This pharmacologically active molecule has various health benefits, such as neuroprotective effects^21^ as well as biological activities relevant for immunosurveillance^22^, hyperuricemia^23^ and hypoxia-induced damage treatment^24^. Accordingly, there has been great interest in this molecule for application in health-promoting products^16^ including for wound healing^3^. Verbascoside was therefore standardized in the extracts accordingly. Verbascoside content was significantly (p < 0.05) noted to be highest in AE3 (Fig. 2C). The glycosidic bond of verbascoside may be degraded during extraction for a longer period of time. Thus, maceration of sea holly leaves for 3 h was determined to be the optimal time for verbascoside-rich extract preparation. Which, the presenting study afforded sea holly extracts with greater verbascoside contents than the previous report (9.58 ± 0.65% w/w) macerated the herb for 3 days in EtOH^25^. Accordingly, the source, herbal preparation practice and the extraction are crucially indicated on the herbal extract’s quality in term of the active content as well as the extractive yields aforementioned above.

### Wound healing effect of the *A. ebracteatus* extracts

Wound healing process is a complex cellular and molecular mechanisms that generally defied in inflammatory, proliferative and maturation phases, which the phases facilitate wound healing are overlap and continuous. Fibroblasts are one of the most recognized cells for skin wound healing process studies. Which, cell migration study is quantified in term of wound closure or wound gap reduction^18^. Wound healing effect of the extracts and its active component was comparatively challenged using a scratch assay. Cell migration is a hallmark of wound repair, and the scratch assay is regarded as a reliable method to study the collective cell migration that determines wound healing activity^18,19^. This assay is therefore widely performed to evaluate herbal extract candidates^26,27^ for wound healing^3,4^.

The cellular safety ranges of the extracts towards fibroblasts were examined. Which, the extracts’ concentration ranges that negatively cause cytotoxicity were wider than verbascoside (Fig. 3A). Thereafter, wound healing efficiency of the extracts in term of cell migration was examined by mean of the scratch assay. Scratch of each well seeded with fibroblasts was created using the pipette tip delineated by the recommended protocol. The long-axial of the tip was perpendicular to the bottom of the well, scratch in a strength line (one direction). Two scratch marks (left and right) was therefore conformally made on each well^19^ to generate the uniformed wound width (0.5 mm). At which, the extracts and verbascoside were monitored at the same selected non-cytotoxic concentrations (20 μg/mL). The extracts and the herbal active component, verbascoside, were weaker than the benchmark FGF (0.1 μg/mL) in wound healing ability (Fig. 3C). The wound healing effect of AE3, the extract with highest verbascoside content, was the best among the extracts. Interestingly, a wound healing proficiency of AE3 was better than its active component verbascoside. The wound healing activity of AE3 should be therefore attributed to synergy between verbascoside and additional phenolic constituents^15^ promoting cell migration that is a critical phase in wound healing^18^. Although the extracts were less potent than FGF, they were proved to establish cutaneous homeostasis following injury and notably accelerated wound healing process. Consequently, the healing mechanism towards extracellular matrix molecules associated in wound healing is worthily to be explored. The extracts’ activity against extracellular matrix degradation enzyme, i.e., MMP-9, the MMP of significant in wound healing process^8^ was examined.

### Inhibitory effect against MMP-9 of the *A. ebracteatus* extracts

Migration of fibroblasts is the crucial step in wound healing process, and is monitorable by the scratch assay^18,19^. The migrating fibroblasts change into myofibroblasts on macrophages stimulation, and gap the wound edges together. Macrophages not only escalate positive outcomes for wound closure, but blunting the inflammations^6^ and upregulating MMP secretions^8^ lessen wound healing quality consequently^3^. Which, MMP-9 is of particular interest studied MMP in the wound healing process^5,8^. Thus, the extracts’ wound healing mechanism in term of an inhibitory effect against MMP-9 was examined.

Cocultures were firstly constructed. Fibroblasts were seeded in 48-well plate for 3 days prior to additional seed of HaCaT cells. The non-cytotoxic concentrations revealed in HaCaT and fibroblast cells (Fig. 3A) were thereafter guided for the safety assessment in the cocultures, which high concentrations were challenged (Fig. 4A). At which, 150 µg/mL was noted as an appointed dose for the extracts and verbascoside further assessments, while 10 µg/mL was for dexamethasone. The activity of the extracts against MMP-9 was consequently examined in cocultures at the non-cytotoxic doses. The extracts were evidenced to protect against UV-induced cellular damaged. Which, the working mechanism was by an inhibitory effect against MMP-9 (Fig. 4B). Notably, the anti-MMP-9 activities of the extracts were significantly (p < 0.001) better than its active constituent, i.e., verbascoside. Thus, the cellular activity of the extracts should also be affected by the additional phenolic constituents in a combination with the major active compound verbascoside. An inhibitory effect against MMP-9 would elevate skin wound healing and suppress the inflammation^6^. Which, a prolonged inflammatory phase and blunt inflammation may interfere with the proliferation and maturation phases delaying wound healing accordingly^7^. In addition, anti-MMP-9 would prevent a scar formation from an event resulted from MMP-9 overexpression as well^5^.

In summary, *A. ebracteatus*, a medicinal herb used in traditional remedies, is proved upon its ethnopharmacological doctrine for skin disorder treatment, i.e., wound healing. The herb exhibited a significant wound healing effect by means of its promotion activity on cell migration as assessed with the scratch assay. The wound healing mechanism of the herb was additionally revealed by its anti-MMP-9 activity as evidenced in cocultures. The herb is therefore applicable to create the appropriated ecosystem for wound healing process with a good cell migration proficiency and less or abrogate scar formation co-contributing for a successful wound healing. In addition, the herb was shown to be a prominent source of verbascoside, that elaborate for a variety of health-promotion products in line with health benefits^21–24^ of this pharmacologically active molecule. The present findings fill in the gap upon the traditionally uses of the herb and pave the way for further study in search of its potential working mechanism in addition to its profound effect on cell migration and anti-MMP-9, which applicable to new potential targets for wound healing as well. Accordingly, verbascoside-rich *A. ebracteatus* could be utilized for the development of innovative skin-care products in regards with its significant prove on chemical and biological profiles. Furthermore, assessment in human volunteers would be encouraged delineates by this presenting results.

## Materials and methods

### Materials

HDF and keratinocyte (HaCaT cells) (ATCC, USA), were used in this study. Cellular safety assessment was preliminary examined with SRB assay^28^. The chemicals for an extraction were of commercial grade, analytical HPLC grade were used for verbascoside analysis, those for cell culture assessments were analytical grade.

### Preparation of the extracts

The leaves of *A. ebracteatus* (AEL) were harvested from the farm located at Nan Province, Thailand in November 2020. Plant access and collection practices were complied with national guidelines and legislation, i.e., Plant variety protection Act (1999) of Thailand, with the right permits and following good academic practice. Permission for collection of plant sample was taken from the farm owner; Mr. Chusilp Sanrattana, with an ID of 03-9000-55-990-056024 for organic agriculture certified by Department of Agriculture, Ministry of Agriculture and Cooperatives. Mr. Nirun Vipunngoun from College of Pharmacy, Rungsit University and Mr. Chusilp Sanrattana, the botanists, had identified the plant material with a consent to harvest and collect the plant sample with the voucher specimen of MKAE11NAN20. The aforementioned voucher specimen was deposited for further reference at Phytocosmetics and Cosmeceuticals Research Group, Mae Fah Luang University, where access is public and available. The leaves were cleansed with water, air dried at the temperature of lower than 50 °C under the shade, ground into powder (< 2 mm), macerated in 95% EtOH with shaking at 150 rpm for 1, 3 and 24 h under an ambient condition with the ratio between plant sample and solvent of 1:10 (w/v). The whole was filtrated and concentrated to dryness to give AE1, AE3 and AE24 extracts, respectively. The extractive yield was calculated. Each extraction was undertaken for more two times.

### Verbascoside analysis

Verbascoside content of each extract was quantified by UPLC (ACQUITY H-Class system) equipped with an ACQUITY UPLC PDA eλ detector using a BEH C18 1.7 µm column (2.1 × 100 mm). The standard verbascoside (Sigma-Aldrich, ≥ 99% HPLC grade) at various concentrations in AcCN was used to prepare calibration curves (r > 0.9995). The analytical condition was made in a gradient mobile phase consisting of AcCN (A) and 3% aq. AcOH (B). The eluent was programmed as follows: 0 min 100% B, 1.5 min 95% B, 3 min 85% B, 5 min 80% B and 8 min 70% B at a flow rate of 0.6 mL/min for a 10 min completing an injection^28^. The maximum absorption wavelength of verbascoside was determined under this analytical condition at 330 nm, at which this wavelength was set for its quantification in the extracts in addition to the determined retention time. The analysis was performed in three cycles.

### Scratch assay

A scratch assay assesses the expansion of HDF population on surfaces was undertaken by the literature method^26,27^. Briefly, the cells cultivated in DMEM (Gibco, USA) supplemented with 10% FBS (Gibco) and penicillin–streptomycin solution (Gibco), were seeded in a culture-insert 4 well plate in µ-dish 35 mm (ibidi GmbH, Gräfelfing, Germany) for 24 h. Scratch of each well was created using the pipette tip. In each well, 2 scratches marks (left and right) were made. The cells were washed with PBS, treated with sample that are a positive control, FGF (100 ng/mL; American Type Culture Collection (ATCC, USA)), the extracts and verbascoside (20 µg/mL, each), and incubated. The gap area was recorded at 24 and 48 h in a comparison with the initial gap size at 0 h. Average of left scratch and right scratch obtained from 6 points per scratch were taken separately, and calculated as the gap area. The migration rate was calculated as following;

Migration (%) = [(Initial gap area – gap area time interval)/Initial gap area] × 100.

The experiment was performed in triplicate.

### Inhibitory effect against MMP-9 in a coculture of HaCaT cells and HDF

Cellular safety of the samples was examined in a coculture model. In short, HDF were seeded in 48-well plate for 3 days prior to additional seed of HaCaT cells seeded onto the HDF and further incubated for 24 h. Thereafter, the cocultures were treated with the samples, i.e., AE1, AE3, AE24, verbascoside and dexamethasone (D4902, Sigma, UK), incubated for 24 h, exposed with UV (UV lamp, Cole-Parmer, USA), i.e., UVA for 15 min (1 J/cm^2^) and UVB for 25 s (30 mJ/cm^2^), incubated for 24 h. Cell viability (%) was monitored with CellTiter-Glo luminance (Promega, USA). Cellular activity of the samples against MMP-9 (ab100610, Abcam, UK) in the coculture system was examined at the noncytotoxic concentrations (150 µg/mL for AE1, AE3, AE24 and verbascoside, 10 µg/mL for dexametasone) in a comparison with the control groups, i.e., vehicle control with UV exposures and vehicle non-UV control^29^.

### Statistical analysis

Data were presented as the means ± SD, a one-way ANOVA test and post-hoc Tukey's Honest test were used to evaluate the difference between groups using the program SPSS version 16.0. The level of significance was at p < 0.05.

## Data Availability

The data supporting this study are available upon a reasonable request to the corresponding author.

## Abbreviations

AcCN: Acetonitrile
AcOH: Acetic acid
AE1: *A. ebracteatus* 1 h extract
AE3: *A. ebracteatus* 3 h extract
AE24: *A. ebracteatus* 24 h extract
AEL: *A. ebracteatus* leaves
ATCC: American Type Culture Collection
DMEM: Dulbecco’s Modified Eagle’s Media
EtOH: Ethanol
FBS: Fetal Bovine Serum
FGF: Fibroblast Growth Factor
h: Hour
HDF: Human Dermal Fibroblasts
MMP: Matrix Metalloproteinases
PBS: Phosphate-Buffered Daline
SRB: Sulforhodamine B
UPLC: Ultra-Performance Liquid Chromatography
UV: Ultraviolet
